# Florivory and pollinator visitation: a cautionary tale

**DOI:** 10.1093/aobpla/plw036

**Published:** 2016-07-11

**Authors:** Kaoru Tsuji, Manpreet K. Dhami, David J.R. Cross, Carolyn P. Rice, Nic H. Romano, Tadashi Fukami

**Affiliations:** 1 Department of Biology, Stanford University, Stanford, CA 94305, USA; 2 Center for Ecological Research, Kyoto University, 2-Hirano, Otsu, Shiga 520-2113, Japan

**Keywords:** Flower, hummingbirds, *Mimulus aurantiacus*, nectar microbes, petal herbivory, stigma closure

## Abstract

Florivory, or damage to flowers by herbivores, can make flowers less attractive to pollinators, potentially resulting in reduced plant fitness. However, not many studies have combined observations with experiments to assess the causal link between florivory and pollination. We conducted field observations at eight sites in northern California, combined with field experiments that involved artificial floral damage, to study the effect of florivory on pollination in the hummingbird-pollinated sticky monkeyflower, *Mimulus aurantiacus*. We used two indicators of pollinator visitation, stigma closure and the presence of microorganisms in floral nectar. The field observations revealed that stigma closure was less frequent in damaged flowers than in intact flowers. In the experiments, however, floral damage did not decrease stigma closure or microbial detection in nectar. Instead, neighbouring flowers were similar for both indicators. These results suggest that the observed negative association between florivory and pollination is not causal and that the location of flowers is more important to pollinator visitation than florivory in these populations of *M. aurantiacus*.

## Abstract (precis)

Floral herbivory can make flowers less attractive to pollinators. To study the effect of floral herbivory on pollination in the hummingbird-pollinated sticky monkeyflower, we conducted field observations and experiments. We used two indicators of pollinator visitation: stigma closure and the presence of microorganisms in floral nectar. The field observations revealed that stigma closure was less frequent in damaged flowers than in intact flowers. In the experiments, however, floral damage did not decrease stigma closure or microbial detection. These results tell a cautionary tale: a negative association between florivory and pollinator visitation can be observed without florivory affecting pollinator visitation.

## Introduction

Floral herbivory can be as widespread as foliar herbivory, but its potential effects on plant fitness have only recently begun to be investigated (McCall and Irwin 2006; Maldonado *et al.* 2015). Florivory often reduces flower size (Strauss and Whittall 2006) and nectar production (Krupnick *et al.* 1999; Strauss and Whittall 2006), both of which may reduce pollinator visitation as many pollinators tend to prefer larger flowers and greater nectar production (Bell 1985; Galen 1989; Kudoh and Whigham 1998; Krupnick *et al.* 1999; Arista and Ortiz 2007; Shumitt 2014). An increasing number of studies suggest that floral damage can indeed decrease pollinator visitation (Karban and Strauss 1993; Pohl *et al.* 2006; Ashman and Penet 2007; Penet *et al.* 2009; Cardel and Koptur 2010; Sõber *et al.* 2010; Cares-Suárez *et al.* 2011), potentially resulting in reduced pollination and plant fitness (Krupnick and Weis 1999; Mothershead and Marquis 2000; Leavitt and Robertson 2006; McCall and Irwin 2006; Strauss and Whittall 2006; Sánchez-Lafuente 2007; Carezza *et al.* 2011).

However, studies on florivory have often used either field observations or experiments, not both (but see examples of using both in McCall 2008, 2010). Combined use of observations and experiments is needed in order to determine (i) if a relationship exists between florivory and pollination in natural populations, through observations, and (ii) if the observed florivory–pollination relationship is causal, through experiments. In this paper, we report a study that examined whether florivory was related to pollinator visitation through a combination of field observations and experiments. We first observed floral damage (primarily to petals) and stigma closure, an indicator of pollination in the sticky monkeyflower, *Mimulus aurantiacus*, at eight sites across an ∼200 km geographic region, to investigate the relationship between florivory and pollinator visitation. The data revealed a negative association. To examine if this association was causal, we then conducted field experiments, in which *M. aurantiacus* flowers were artificially damaged and two indicators of pollinator visitation recorded, stigma closure and the presence of microorganisms in nectar.

## Methods

### Species description

*Mimulus aurantiacus* is a hummingbird-pollinated perennial and self-compatible shrub native to California and Oregon (Fetscher and Kohn 1999; Vannette *et al.* 2013). The stigma of *M. aurantiacus* closes upon contact and stays closed if much pollen is received, but reopens if no or little pollen is received (Fetscher and Kohn 1999). For this reason, stigma closure can be used as an indicator of pollinator visitation in this species (Peay *et al.* 2012; Vannette *et al.* 2013). Furthermore, many of the microorganisms that are found in *M. aurantiacus* nectar are introduced to flowers primarily via hummingbirds (Belisle *et al.* 2012). Thus, the presence of microorganisms in floral nectar can also be used as an indicator of pollinator visitation. To estimate the age of the flowers, we used the condition of stamens as a proxy. *Mimulus* flowers have four stamens per flower, which dehisce and deteriorate as the flowers age. Young flowers (most likely 1–2 days old) have undehisced yellow stamens, while middle-aged flowers (typically 3–5 days old) have orange-brown dehisced stamens. Older flowers (typically 6–8 days old) have dark brown and shrunken or degenerate stamens. Stamen condition based on this distinction was used to categorize flowers into estimated age groups.

### Field observations

**Figure 1.**
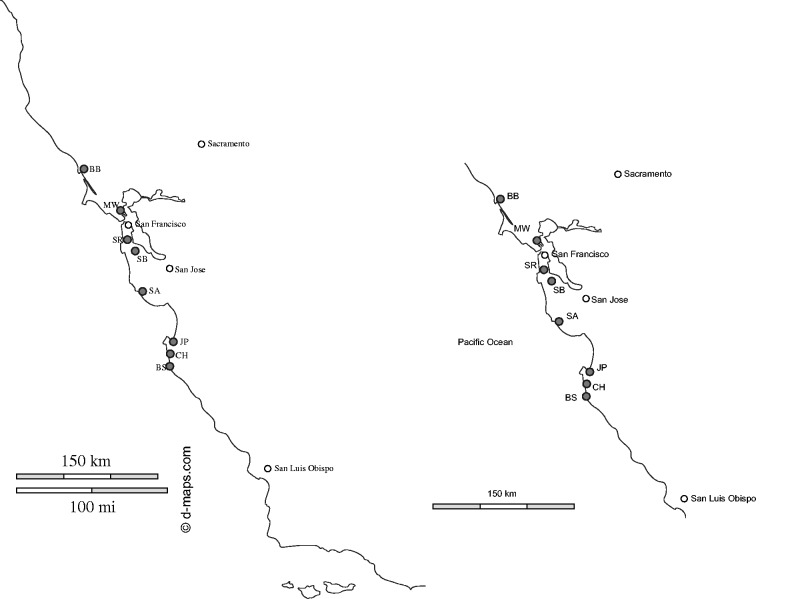
Site of observations (see Table 1 for coordinates).

**Figure 2.**
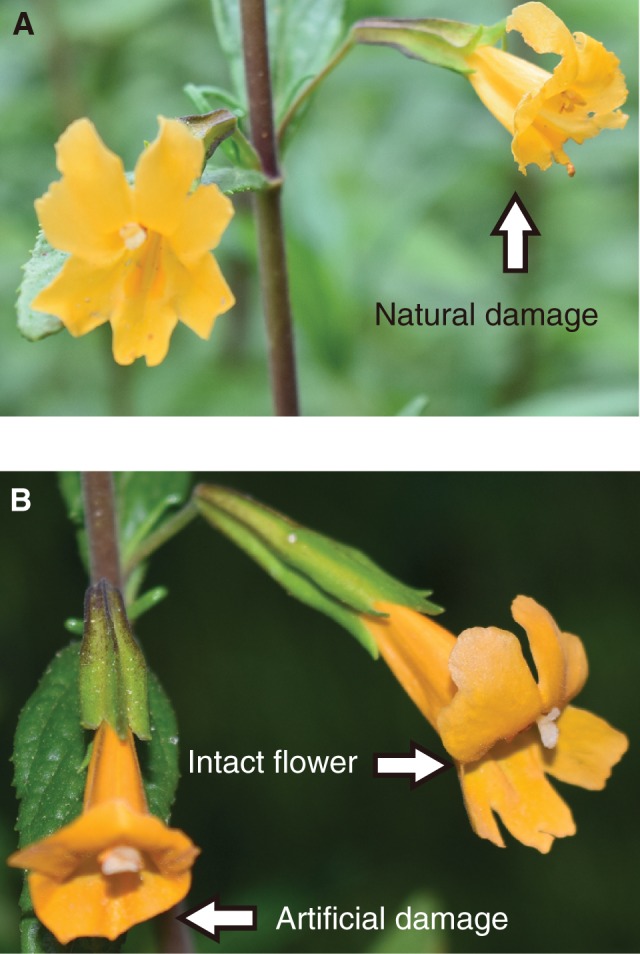
Photographs showing (A) a flower with natural damage by florivorous insects and (B) an example of paired flowers, with one intact and one experimentally damaged. Photo credit: P. Garvey and M. Dhami.

We recorded stigma closure (open or closed), flower damage (observed primarily on petals, and not the rest of the floral organs, including the stigma) and the age of flowers (young, middle-aged or old) from a total of 500 haphazardly selected flowers on 60 individuals at 8 sites in northern California (Fig. 1 and Table 1), between 30 June and 16 July 2015. We recorded the extent of floral damage for each flower we observed (i.e. intact, partially damaged, half damaged, heavily damaged), but we did not find any significant effect of the floral damage extent on pollination, so we focused on the presence or the absence of damage (i.e. intact or damaged) in the analyses presented in this paper. For data collection at each site, we haphazardly chose plants along roads and selected 6–15 flowers from each of the plants. We did not directly confirm that all of the damage on each flower we sampled for this study was actually caused by florivores. However, our extensive observations at one site (Jasper Ridge Biological Preserve of Stanford University) indicated that many, if not all, instances of floral damage, which left holes and bits of variable sizes (Fig. 2A), were caused by insect florivores, such as katydids, grasshoppers and lepidopteran larvae.

**Table 1.** Observed number of plants and flowers at eight sites.

Site	Coordinates	No. of observed plants	No. of observed flowers
BB	38.20, 123.02, 25	13	113
MW	37.53, 122.34, 184	10	98
SR	37.37, 122.27, 216	6	47
SB	37.29, 122.21, 335	12	93
SA	37.05, 122.15, 136	4	32
JP	36.34, 121.51, 196	4	31
CH	36.24, 121.54, 51	6	47
BS	36.20, 121.53, 106	5	39
Total		60	500

The data were analysed using R (3.11 version, The R foundation for Statistical Computing Platform). We used a generalized linear mixed model (GLMM) with binomial distribution and a logistic function in lme4 package, and used the likelihood ratio test in order to determine whether stigma closure was significantly associated with flower damage and age. In the model, we used stigma closure as the response variable, flower age, flower damage, and the interaction of the age and the damage as fixed predictors, and shrub individuals and sites as random effects. In addition, we also used a similar GLMM to determine whether flower damage significantly differed among individuals and among sites. In this model, we used flower damage as the response variable, flower age as fixed predictors, and shrub individual and site as random effects. Finally, we also used a regression analysis to test whether the overall proportion of flowers that had a closed stigma at a site was significantly correlated with the frequency of flowers that had natural damage at the matching site. For this regression, we focused on old flowers.

### Field experiments

We marked a total of 236 pairs of flowers that were located close to each other (within 30 cm) on a total of 83 plants and artificially damaged on one of each of the paired flowers. Artificial florivory was intended to mimic a severe level of naturally observed florivory on petals (Fig. 2B). This experiment was conducted at site SB (Fig. 1) and at a common garden at the Stock Farm plant growth facility on the Stanford University campus in Stanford, CA, USA. At the SB site, we used young flowers for this experiment, conducted from 24 to 28 July 2015. At the common garden, the experiment was repeated nine times from 4 to 28 August 2015, using both young and middle-aged flowers. At both sites, we observed hummingbirds (*Calypte anna*) frequently visiting *M. aurantiacus* flowers.

For each pair of flowers, 4 days after making artificial damage, we recorded stigma closure and additional natural damage on the flowers, and then collected the flowers. From each of the collected flowers, we extracted nectar using a 10-µL microcapillary tube and delivered the nectar into 40 µL of sterile water. The diluted nectar samples were further diluted and plated on yeast malt agar (YMA; Difco, Sparks, MD, USA) supplemented with 100 mg mL^−1^ of the antibacterial chloramphenicol. After 5 days of incubation at 25°C, we checked for the presence or the absence of microbial colonies on the agar plates.

Chloramphenicol was used so as to focus on the presence of yeast, such as *Metschnikowia reukaufii*, rather than bacteria, in nectar. In *M. aurantiacus*, we have previously found that yeasts appeared more dependent on hummingbirds for nectar colonization than bacteria (Belisle *et al.* 2012). The presence of yeasts in nectar therefore likely serves as a better indicator of pollinator visitation than that of bacteria. However, our previous work with molecular identification of colonies has also indicated that some bacterial taxa may be capable of forming colonies on chloramphenicol-supplemented YMA and that these bacterial colonies tend to be distinctly smaller than yeast colonies. For this reason, we also analysed the presence of large colonies, but the results were almost identical regardless of whether we considered all colonies or only large colonies. In this paper, we report results for all colonies.

Data on the frequency of flowers from which microbial colonies were detected were analysed by *χ*^2^ test and Fisher’s exact test in *R*, in order to determine if artificial damage affected the frequency of stigma closure or the occurrence of microorganisms in nectar. In addition, we also used *χ*^2^ test and Fisher's exact test to determine whether the paired flowers were more similar to each other at the end of the experiment than expected by chance in their pollination status. Furthermore, we used GLMM with binomial distribution and a logistic function in lme4 package, and used the likelihood ratio test in order to determine whether stigma closure or microbial detection was significantly associated with flower damage. In this model, we used stigma closure or microbial detection as the response variable, flower age, flower damage and the interaction of age and damage as fixed predictors, and pair ID and site as random effects. For these analyses, we excluded pairs in which any natural damage was observed on the initially intact flower at the time of data collection. The results were qualitatively the same, however, when we included these pairs in the analyses.

## Results

### Field observations

**Figure 3.**
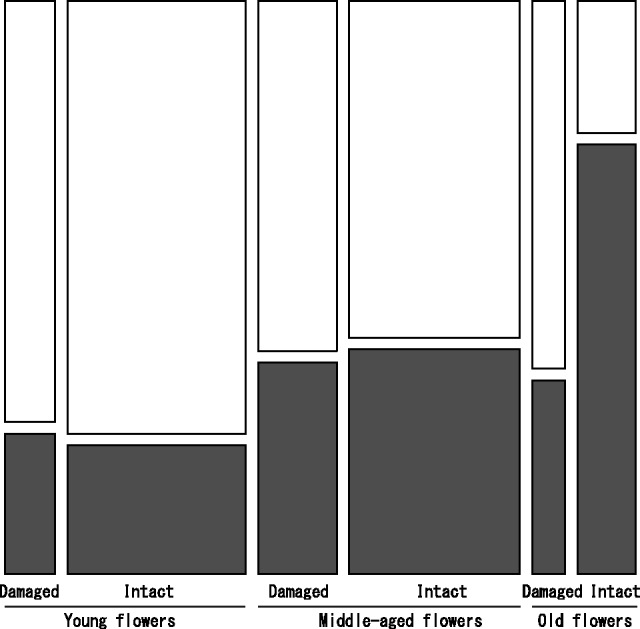
Mosaic plot summarizing field observations on the frequency of stigma closure of intact and damaged flowers depending on flower age. Black bars represent flowers with closed stigmas, and white bars represent flowers with open stigma. The area of the tiles is proportional to the number of observations in the corresponding category of flower age and damage status (total number of flowers observed = 500).

Stigma closure was significantly related to flower age, flower damage, their interaction, shrub individual and observation site (Table 2). Frequency of stigma closure increased significantly with increasing flower age and was significantly lower in damaged flowers (34.5 %) than in intact flowers (76.4 %) when old flowers were observed (Fig. 3). Similarly, the likelihood of observing flower damage itself varied significantly with flower age, shrub individual and observation site [see **Supporting Information – Table 1 and Fig. 1**]). At the site scale, the frequency of stigma closure was negatively correlated with the proportion of floral damage (*t *= −3.39, *P* = 0.019 [see **Supporting Information – Fig. 2**]).

**Table 2.** Analytical results of field observations using the likelihood ratio test.

Predictor	*df*	Likelihood	*P* value
Flower age	2	68.13	<0.0001
Florivorous damage	2	13.22	0.001
Age × damage	1	44.73	<0.0001
Shrub individuals	1	5.33	0.02
Sites	1	13.68	0.0002

### Field experiments

Floral damage did not significantly decrease stigma closure or the detection of microbes in nectar (stigma: odds ratio* *= 0.36, *df* = 1, *P* = 0.55; microbes: odds ratio = 0.19, *df* = 1, *P* = 0.66, Tables 3 and 4). This pattern was observed at both sites (stigma: odds ratio = 0.42, 0, *df* = 1, 1, *P* = 0.52, 1; microbes: odds ratio = 0, 0.56, *df* = 1, 1, *P* = 1, 0.45, at the SB site and the Stock Farm site, respectively, [see **Supporting Information – Tables 2–5**].

**Table 3.** Stigma closure of intact and experimentally damaged flowers.

	Damaged flowers	Intact flowers
Closed stigma	94 (46 %)	87 (42 %)
Open stigma	111 (54 %)	118 (58 %)
Total	205 (100 %)	205 (100 %)

**Table 4.** Microbial detection in nectar from intact and experimentally damaged flowers with open or close stigmas.

	Damaged flowers	Intact flowers
Detected	74 (44 %)	79 (47 %)
Undetected	93 (56 %)	88 (53 %)
Total	167 (100 %)	167 (100 %)

However, paired flowers were similar in both stigma closure and the presence of microbes. That is, if a flower had a closed stigma, it was significantly more likely that the paired flower also had a closed, than open, stigma (odds ratio = 9.05, *df *= 1, *P* = 0.0026, Table 5). Likewise, if microbes were detected from a flower, it was significantly likely that they were also detected from the paired flower (odds ratio = 49.26, *df *= 1, *P* < 0.0001, Table 6). Consistent with these results, the GLMM also indicated that stigma closure and microbial detection were significantly related to pair ID, but not to flower damage [see **Supporting Information – Tables 10 and 11**. When analysed separately for the two sites, the pattern was significant for stigma closure at SB (odds ratio = 12.3, *df* = 1, *P* = 0.0005 [see **Supporting Information – Table 6**]), but not at Stock Farm (odds ratio* *= 0, *df* = 1, *P* = 1 [see **Supporting Information – Table 7**]), and it was significant for microbial detection at Stock Farm (odds ratio = 7.9, *P* = 0.005 [see **Supporting Information – Table 9**]), but not at SB (odds ratio = 1.6, *df* = 1, *P* = 0.21 [see **Supporting Information – Table 8**]). These differences between the two sites were likely due to differences in sample size, which affected statistical power.

**Table 5.** Stigma closure in paired flowers in the field experiments.

	Damaged flowers with close stigma	Damaged flowers with open stigma
Intact flowers with close stigma	51 (54 %)	36 (32 %)
Intact flowers with open stigma	43 (46 %)	75 (68 %)
Total	94 (100 %)	111 (100 %)

**Table 6.** Microbial detection from nectar in paired flowers in the field experiments.

	Damaged flowers from which microbes were detected	Damaged flowers from which microbes were not detected
Intact flowers from which microbes were detected	58 (78 %)	21 (23 %)
Intact flowers from which microbes were not detected	16 (22 %)	72 (77 %)
Total	74 (100 %)	93 (100 %)

## Discussion

Taken together, our results tell a cautionary tale: a strong negative association between florivory and pollinator visitation can be observed without florivory affecting pollinator visitation. In the field observations, frequency of stigma closure was clearly lower in damaged flowers than in intact flowers (Fig. 3). However, our experiments yielded no evidence of florivory decreasing pollinator attraction (Tables 3 and 4). Artificial florivory might differ from natural florivory, as in reports on foliar herbivory (Lehtilä and Boalt 2004), particularly with regards to chemical change induced by florivory (Zangerl and Berenbaum 2009; Lucas-Barbosa *et al.* 2013). Nonetheless, our results suggest that reduction in petal size by florivory is unlikely to affect pollinator attraction in the populations of *M. aurantiacus* we studied, at least at the spatial scale considered here.

Instead, we found that paired flowers were strongly more similar in both stigma closure and the presence or the absence of microbes in nectar than expected by chance (Tables 5 and 6). This result suggests that the position of flowers may be more important for pollinator visitation than florivory. The reason for the observed negative relationship between florivory and pollinator attraction remains unclear, but it may have been caused by differences in the locations where florivorous insects are common and those where hummingbirds preferred to visit flowers. In other words, it is possible that, where florivores were abundant, pollinators were not and *vice versa* [see **Supporting Information – Fig. 2**], without any causal relationship between the two groups. In fact, we found that individuals and sites were both significant predictors of stigma closure (Table 2), suggesting that spatial differences in florivore vs. pollinator availability may have existed at both the individual and site scales. This possibility seems plausible because hummingbirds often forage in a spatially limited local area, particularly when they are territorial (Lima 1991; Eberhard and Ewald 1994), and florivores can also be highly patchy in their spatial distribution at small scales (Loxdale and Lushai 1999).

In this study, we only used stigma closure and microbial presence as proxies for pollination. However, pollinator attraction can have different effects on male fitness (pollen dispersal) and female fitness (seed set) (Krupnick and Weis 1999), and different microbes can differently affect pollinator attraction and subsequent seed set (Vannette *et al.* 2013). To quantify more directly the potential effect of florivory on plant fitness via pollinator attraction, seed set and pollen export should be measured. The effects of different microbial species on pollination should also be studied. Moreover, we did not directly quantify spatial variation in the abundance of florivores and pollinators, yet our results suggest that such variation may underlie the negative relationship between florivory and pollination. Finally, although we found no causal relationship between florivory and pollinator visitation in this study, it would be useful to apply artificial floral damage at different spatial scales (e.g. using paired plants or patches of plants, as opposed to paired flowers within plants) to determine the scale at which florivory might potentially affect foraging decisions by hummingbirds.

## Conclusions

Our results suggest that the observed negative association between florivory and pollination is not causal and that the location of flowers may be more important to pollinator visitation than florivory in these populations of *M. aurantiacus*. Based on these findings, we suggest that spatial variation in florivores and pollinators should be taken into account in order to understand potential florivory effects on pollination.

## Sources of Funding

Financial support was provided by the National Science Foundation (award number: DEB 1149600), JSPS Postdoctoral Fellowship (25451) and the VPUE summer research program, the Department of Biology and the Terman Fellowship of Stanford University.

## Contributions by the Authors

K.T. conceived the study and K.T., M.K.D. and T.F. designed the experiments. K.T., M.K.D., D.J.R., C.P.R., N.H.R. and T.F. performed the experiments. K.T. and T.F. analyzed the data and wrote the manuscript. All authors contributed to revising the manuscript.

## Conflicts of Interest Statement

None declared.

## Supplementary Material

Supplementary Data

## Supporting Information

The following additional information is available in the online version of this article —

**Figure 1**. Mosaic plot of the frequency of damaged flowers in field observations. Each bar represents a plant individual. The black portion of each bar represents the proportion of flowers with damaged flowers, and the white portion represents intact flowers. The area of the tiles is proportional to the number of observations in the corresponding category (total number of flowers observed = 500).

**Figure 2**. Relationship between the proportion of damaged flowers and the proportion of flowers that had a closed stigma. Each data point represents a site.

**Table 1.** Analytical results of field observations using the likelihood ratio test.

**Table 2.** Stigma closure of intact and experimentally damaged flowers at the SB site.

**Table 3.** Stigma closure of intact and experimentally damaged flowers at the Stock Farm site.

**Table 4.** Microbial detection in nectar from intact and experimentally damaged flowers with open or closed stigmas at the SB site.

**Table 5.** Microbial detection in nectar from intact and experimentally damaged flowers with open and closed stigmas at the Stock Farm site.

**Table 6.** Stigma closure in paired flowers in the field experiments at the SB site.

**Table 7.** Stigma closure in paired flowers in the field experiments at the Stock Farm site.

**Table 8.** Microbial detection from nectar in paired flowers in the field experiments at the SB site.

**Table 9.** Microbial detection from nectar in paired flowers in the field experiments at the Stock Farm site.

**Table 10.** Analytical results of field experiment for stigma closure using Likelihood ratio test.

**Table 11.** Analytical results of field experiment for microbial detection using Likelihood ratio test.
