# Supplementary material for: Florivory and pollinator visitation: a cautionary tale
Source: AoB Plants. 2016 Jul 11;8:plw036. doi: 10.1093/aobpla/plw036 (PMC4940504; doi:10.1093/aobpla/plw036)
Supplement: Supplementary Data [file supp_8_plw036_index.html]

Florivory and pollinator visitation: a cautionary tale — Supplementary Data 

# Florivory and pollinator visitation: a cautionary tale

## Supplementary Data

files

- Supplementary Data - docx file
- Supplementary Data - docx file
