# Supplementary material for: Florivory and pollinator visitation: a cautionary tale
Source: AoB Plants. 2016 Jul 11;8:plw036. doi: 10.1093/aobpla/plw036 (PMC4940504; doi:10.1093/aobpla/plw036)

**Fig. S1**

Mosaic plot of the frequency of damaged flowers in field observations. Each bar represents a plant individual. The black portion of each bar represents the proportion of flowers with damaged flowers, and the white portion represents intact flowers. The area of the tiles is proportional to the number of observations in the corresponding category (total number of flowers observed = 500).


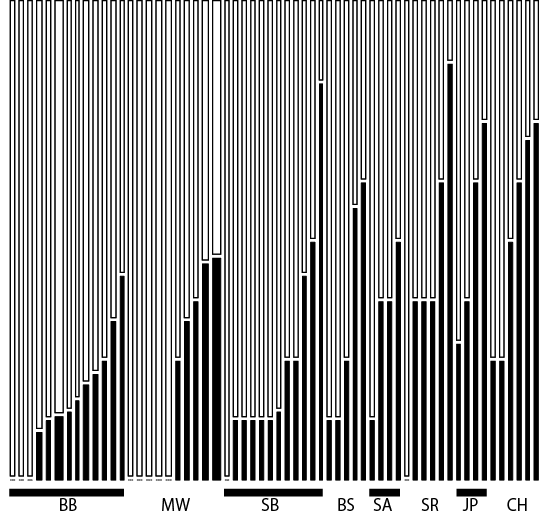


**Fig. S2**

Relationship between the proportion of damaged flowers and the proportion of flowers that had a closed stigma. Each data point represents a site.


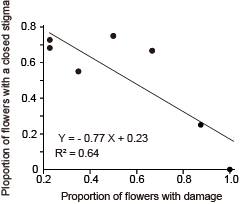

Supplement: Supplementary Data [file supp_plw036_aobplants-16025-s02.docx]
