# Supplementary material for: Florivory and pollinator visitation: a cautionary tale
Source: AoB Plants. 2016 Jul 11;8:plw036. doi: 10.1093/aobpla/plw036 (PMC4940504; doi:10.1093/aobpla/plw036)
Supplement: Supplementary Data [file supp_plw036_aobplants-16025-s03.docx]

**Table S1.** Analytical results of field observations using Likelihood ratio test.

| Predictor | *df* | Likelihood | *p* - value |
| --- | --- | --- | --- |
| Flower age | 1 | 19.27 | < 0.0001 |
| Shrub individuals | 1 | 4.89 | 0.027 |
| Sites | 1 | 14.53 | 0.0001 |

**Table S2.** Stigma closure of intact and experimentally damaged flowers at the SB site.

|  | Damaged flowers | Intact flowers |
| --- | --- | --- |
| Closed stigma | 64 (53%) | 58 (48%) |
| Open stigma | 56 (47%) | 62 (52%) |
| Total | 120 (100%) | 120 (100%) |

**Table S3.** Stigma closure of intact and experimentally damaged flowers at the Stock Farm site.

|  | Damaged flowers | Intact flowers |
| --- | --- | --- |
| Closed stigma | 30 (35%) | 29 (34%) |
| Open stigma | 55 (65%) | 56 (66%) |
| Total | 85 (100%) | 85 (100%) |

**Table S4.** Microbial detection in nectar from intact and experimentally damaged flowers with open or close stigmas at the SB site.

|  | Damaged flowers | Intact flowers |
| --- | --- | --- |
| Detected | 13 (15%) | 13 (15%) |
| Undetected | 72 (85%) | 72 (85%) |
| Total | 85 (100%) | 85 (100%) |

**Table S5.** Microbial detection in nectar from intact and experimentally damaged flowers with open or close stigmas at the Stock Farm site.

|  | Damaged flowers | Intact flowers |
| --- | --- | --- |
| Detected | 61 (74%) | 66 (80%) |
| Undetected | 21 (26%) | 16 (20%) |
| Total | 82 (100%) | 82 (100%) |

**Table S6.** Stigma closure in paired flowers in the field experiments at the SB site.

|  | Damaged flowers with close stigma | Damaged flowers with open stigma |
| --- | --- | --- |
| Intact flowers with close stigma | 41 (64%) | 17 (30%) |
| Intact flowers with open stigma | 23 (36%) | 39 (70%) |
| Total | 64 (100%) | 56 (100%) |

**Table S7.** Stigma closure in paired flowers in the field experiments at the Stock Farm site.

|  | Damaged flowers with close stigma | Damaged flowers with open stigma |
| --- | --- | --- |
| Intact flowers with close stigma | 10 (33%) | 19 (35%) |
| Intact flowers with open stigma | 20 (67%) | 36 (65%) |
| Total | 30 (100%) | 55 (100%) |

**Table S8.** Microbial detection from nectar in paired flowers in the field experiments at the SB site.

|  | Damaged flowers from which microbes were detected | Intact flowers from which microbes were not detected |
| --- | --- | --- |
| Intact flowers from which microbes were detected | 4 (31%) | 9 (13%) |
| Intact flowers from which microbes were not detected | 9 (69%) | 63 (88%) |
| Total | 13 (100%) | 72 (100%) |

**Table S9.** Microbial detection from nectar in paired flowers in the field experiments at the Stock Farm site.

|  | Damaged flowers from which microbes were detected | Intact flowers from which microbes were not detected |
| --- | --- | --- |
| Intact flowers from which microbes were detected | 54 (89%) | 12 (57%) |
| Intact flowers from which microbes were not detected | 7 (11%) | 9 (43%) |
| Total | 61 (100%) | 21 (100%) |

**Table S10.** Analytical results of field experiment for stigma closure using Likelihood ratio test.

| Predictor | *df* | Likelihood | *p* - value |
| --- | --- | --- | --- |
| Flower age | 2 | 0.72 | 0.7 |
| Artificial damage | 2 | 3.67 | 0.17 |
| Age*Artificial damage | 1 | 0.15 | 0.7 |
| Flower pair ID | 1 | 4.82 | 0.03 |
| Sites | 1 | -1.5e^-7^ | 1 |

**Table S11.** Analytical results of field experiment for microbial detection using Likelihood ratio test.

| Predictor | *df* | Likelihood | *p* - value |
| --- | --- | --- | --- |
| Flower age | 2 | 8.77 | 0.01 |
| Artificial damage | 2 | 0.91 | 0.63 |
| Age*Artificial damage | 1 | 0.28 | 0.60 |
| Flower pair ID | 1 | 11.33 | 0.0008 |
| Sites | 1 | -4.5e^-6^ | 1 |
